# Tennis in the heat: a panel discussion

**DOI:** 10.17159/2078-516X/2026/v38i1a24716

**Published:** 2026-01-15

**Authors:** BM Pluim, O Jay, J Alsma, HAM Daanen, TS Ellenbecker, KA Stroia, B Hainline

**Affiliations:** 1 Section Sports Medicine, Faculty of Health Sciences, University of Pretoria, Pretoria, South Africa; 2 Amsterdam Collaboration on Health and Safety in Sports (ACHSS), IOC Research Center of Excellence, Amsterdam, The Netherlands; 3 Medical Department, Royal Netherlands Lawn Tennis Association (KNLTB), Amstelveen, The Netherlands; 4 Heat and Health Research Centre, Faculty of Medicine and Health, University of Sydney, NSW 2006, Australia; 5 Erasmus University Medical Center, Erasmus MC, Rotterdam, The Netherlands; 6 Department Human Movement Sciences, Faculty of Behavioural and Movement Sciences, Amsterdam, The Netherlands; 7 ATP Tour Inc, 42 East Coast Drive, Atlantic Beach, FL 32233, USA; 8 Performance Health, Women’s Tennis Association, St. Petersburg, FL, USA; 9 ITF Sport Science & Medicine Commission, London, UK; 10 New York University Grossman School of Medicine, New York, NY, USA

**Keywords:** heat stress, cooling, acclimatisation, player safety, thermophysiology

## Abstract

Tennis is played globally across diverse climates and surfaces, exposing athletes to variable levels of environmental heat stress. With rising global temperatures and more frequent heat events, protecting players’ health has become a major priority in the sport. This panel discussion aimed to synthesize current evidence and expert perspectives on measuring, managing, and mitigating heat stress in tennis, with a focus on harmonizing policies across governing bodies and player groups. Experts from sport science, medicine, and tournament operations reviewed recent advances in heat measurement tools, including the limitations of the Wet Bulb Globe Temperature (WBGT) and the emergence of tennis-specific heat stress models. Evidence-based cooling strategies—such as ice towels, shaded recovery, and cold-water immersion—were discussed alongside differentiated policy needs for men, women, juniors, seniors, and wheelchair athletes. The discussion further highlighted challenges in achieving effective heat acclimatisation within professional travel schedules and underscored the importance of proactive medical readiness and player education. Enhanced consistency in heat policies, improved access to cooling resources, and continued collaboration between scientists and governing bodies are essential to safeguard player health and performance under increasing environmental heat stress.

Tennis is played worldwide in a variety of climates and on different surfaces, exposing athletes to a wide range of environmental heat stress. With global warming intensifying and heat events becoming more extreme, protecting players’ health has become an evolving and significant priority in the sport. This issue is further complicated by the sport’s diverse populations—including men, women, juniors, seniors, master athletes, and wheelchair players—each with unique physiological characteristics, match formats, and competitive demands.

Across the professional game, heat policies continue to evolve among the various governing bodies. The Association of Tennis Professionals (ATP) Tour (men) currently lacks a formalised heat rule. However, the ATP has announced that a formal heat rule will be implemented from 1^st^ January 2026. In contrast, the International Tennis Federation (ITF) has already established defined temperature thresholds across all divisions—men’s, women’s, juniors, and masters. ^[1–3]^ The Women’s Tennis Association (WTA) applies its own policy for women’s competition,^[4]^ while ITF wheelchair tennis enforces stricter limits to address the unique thermoregulatory challenges faced by these athletes.^[5]^

At the Grand Slam level, three of the four majors— Australian Open, Wimbledon, and the US Open—have implemented tournament-specific policies, with Roland Garros following the ATP, WTA and ITF.^[6–8]^ Collectively, these approaches reflect an ongoing evolution toward best practice, though continued coordination could enhance consistency and player safety worldwide.

Implementation also varies depending on available medical and infrastructure resources. While some tournaments have comprehensive medical coverage and access to cooling facilities such as shaded areas, fans, and ice baths, others rely primarily on physiotherapists or have limited access to on-site cooling solutions. Strengthening these resources remains an area of shared focus to support the timely recognition and management of heat-related conditions, from cramps and exhaustion to exertional heat stroke.

Against this backdrop, the tennis community continues to enhance its understanding of heat measurement and monitoring, cooling strategies, policy development across player groups, and acclimatisation and medical preparedness—key themes discussed in the upcoming panel session.

This panel brings together experts from sport science, medicine, and tournament operations to share evidence-based insights and promote collaborative approaches for measuring, managing, and mitigating heat stress in tennis—working toward a harmonised framework that prioritises player health, fairness, and performance in an era of increasing environmental challenge. Prof Babette Pluim, Chief Medical Officer of the Royal Netherlands Lawn Tennis Association, facilitates the discussion.

Three of the six panellists—Ollie Jay, Jelmer Alsma, and Hein Daanen—previously presented on this topic during a keynote session at the 2025 Society for Tennis Medicine and Science (STMS) World Congress in Amsterdam, Netherlands, where they shared the latest research and models for heat assessment and management in tennis. All six panellists attended the Congress, with the remaining three—Todd Ellenbecker (ATP), Kathleen Stroia (WTA), and Brian Hainline (ITF)— participating as representatives of the sport’s governing bodies. This unique collaboration of scientific and organisational expertise presents a valuable opportunity to strengthen alignment between research, policy, and on-court practice.

**1. Measuring heat stress in tennis:** **What is the best way for tournaments to assess dangerous heat conditions? Is Wet Bulb Globe Temperature (WBGT) still considered the gold standard, or are better tools emerging?**

**Ollie Jay:** It is important to recognise that the WBGT was initially designed for US Army trainees in hot, humid conditions, so its application in tennis has notable limitations. While WBGT has been useful, it does not account for the unique physical and clothing demands of tennis, nor are its thresholds tennis-specific. The Australian Open, working with the University of Sydney, has pioneered a tennis-specific heat stress scale. This system employs measures of air temperature, mean radiant temperature, wind speed, and humidity assessed courtside, then uses a model tailored to tennis players’ metabolic rates and clothing to estimate risks such as hyperthermia and dehydration. The outcome is a straightforward five-point risk scale: level 1 means play continues as normal, levels 2 to 4 introduce progressively stricter heat-reducing interventions, and level 5 calls for suspension of play. This approach provides a more precise, sport-specific assessment of heat danger for players.

**Jelmer Alsma:** The WBGT is the most widely used standard for assessing environmental heat stress. It integrates temperature, humidity, radiation, and air movement into an absolute index. A higher WBGT indicates an increased risk, but a “normal” value does not eliminate the possibility of severe heat stress in insufficiently acclimatised individuals, since factors like fitness, level of hydration, and acclimatisation strongly affect individual strain. Systems such as Environmental Measurement Units (EMUs) extend WBGT by combining real-time court-side measurements with physiological models, producing actionable risk scores. Additionally, resting heart rate (RHR) and heart rate variability (HRV) can change with heat acclimatisation. They may serve as indicators, but they are not yet validated as reliable measures of full adaptation. The most promising future approach would combine advanced systems such as EMU and personal wearable data (e.g. HRV, RHR) to assess individual heat stress and accompanying risks.

**Hein Daanen**: Temperature alone is insufficient to estimate heat strain, since solar radiation, humidity, and wind speed also play a role. WBGT is standardised by the International Standardisation Organisation (ISO) and is widely used in the field of sports and the military, with established thresholds. While not perfect, it provides a reliable first indicator of heat strain. More advanced thermal models, such as the Predicted Heat Strain standard, may provide additional insights when WBGT thresholds are exceeded.

**Todd Ellenbecker:** From a practical standpoint, WBGT is still commonly used as it has the purported advantage of taking into consideration the radiant energy from the sun, environmental temperature and humidity into a single metric, with established cut-offs for modifying or suspending play. Portable handheld devices, while imperfect, provide practical courtside estimates of WBGT, air temperature, humidity and wind speed, enabling healthcare professionals to monitor heat stress in real time. The use of internet-based international temperature and mapping sites can also provide both real-time and retrospective analysis of thermal stress (WBGT, humidity, temperature, air quality, etc) and provide an adjunct to direct onsite measurement.

**Kathleen Stroia:** The WBGT remains the gold standard for assessing on-court heat stress, as it incorporates temperature, humidity, radiant heat, and wind speed, with strong validation in sport and military contexts. The current consensus recommends continuing to rely on WBGT, supported by regular monitoring—at least 3 times daily (in accordance with the Tennis Extreme Weather Conditions rule, taking into account tournament scheduling and operations, fairness of play, health and safety), and ideally every 2 hours— while exploring newer technologies and models for comparison and research. Real-time WBGT monitoring, when available (portable and affordable for the sport of tennis), and considering or implementing corresponding appropriate responsive measures can inform decision-making and enhance player safety.

**Brian Hainline:** The WBGT remains the gold standard for assessing environmental heat stress because it integrates air temperature, humidity, radiant heat, and wind speed. However, most tournaments lack access to WBGT devices. The Heat Index, though less precise, can be widely adopted as an accessible proxy, particularly when local meteorological data are reliable. Its limitation lies in its inability to reflect on-court microclimates, where surface temperature and direct solar load may differ substantially. Ultimately, accurate measurement must be coupled with education for players, coaches, and families on proactive heat mitigation—recognising early signs of heat illness, optimising hydration, and employing pre-emptive cooling and recovery strategies.

**2. Cooling strategies:** **During matches in hot environments, what are the most effective cooling methods players can safely use?**

**Ollie Jay:** The most effective cooling strategies during tennis matches should be grounded in sound scientific evidence and tailored to the specific environmental conditions (such as hot/humid or hot/dry climates) and the intensity of play. While some methods provide perceptual comfort, only those that significantly lower core temperature truly reduce health risks for athletes.^[9]^ Ice towels, consisting of around 3kg of crushed ice in a damp towel applied during breaks, have been shown to decrease core temperature by approximately 0.5°C over a simulated four-set match, regardless of whether conditions mimic the Australian Open (45°C, low humidity) or the US

Open (36°C, high humidity).^[10,11]^ In humid environments, applying water to the skin and facilitating evaporation with courtside fans also helps, though this is less effective in dry conditions.^[10]^ Drinking cold water can make players feel cooler but does little to lower core temperature, and ingesting ice slurry before play offers only short-lived benefits.^[12]^

**Jelmer Alsma:** In tennis, cooling strategies can be applied before, during, and after play. Pre-cooling methods, such as wearing ice vests or ice caps during warm-up, help lower the body’s initial temperature and delay heat strain. Per-cooling options are limited to changeovers and set breaks and should be rapid and non-disruptive to the match flow (e.g., not impairing grip or movement). Players benefit from sitting in the shade, using fans or mist-fans, and applying cold towels, cold packs, or ice caps. Drinking cold fluids, including ice slurries, is an effective cooling method, but should be practised to avoid gastrointestinal discomfort. Post-cooling is especially important for recovery. Studies show that coldwater immersion lowers core temperature faster than passive recovery and reduces delayed-onset muscle soreness. Coldwater immersion can also speed the return of performance markers. Cold showers are a practical and widely accessible alternative.

**Hein Daanen**: In tennis, breaks can be used to cool off. The best approach combines internal and external cooling: cold drinks and, for instance, a wet towel around the neck. Cooling down during the game can be difficult, but choosing the right clothes can make a big difference. Tight-fitting clothing (in direct contact with the skin) may benefit from reflective dyes or coating (only when the mesh is not too tightly woven). Wearing a hat can help reflect the sun’s rays and reduce heat strain.

**Todd Ellenbecker:** In professional tennis, a combination of strategies has proven effective during matches with only limited research on their efficacy in tennis-specific conditions. The use of ice towels, also known as “ice sausages”, worn around the neck and placed in the groin area during changeovers in tennis, coupled with air movement with fans or blowers, and shading from umbrellas, attempts to provide cooling, air flow and minimise the effects from the sun on players. It is important to note that a major challenge in tennis is the relatively short time (60 to 90 seconds) that players rest between games and sets. As a result, these interventions have only a short period in which they can meaningfully lower core temperature in elite tennis players. Additionally, players have not responded favourably to cooling devices on the hands, given the delicate interface with the hand and racquet immediately after application and its effect on “feel” and stiffness. Continued study is necessary to provide evidence-based guidance in real on-court applications of tennis players to advance our understanding and ultimately protect and enhance player comfort and performance in hot thermal environments.

**Kathleen Stroia:** Cold-water immersion remains the gold standard for rapidly lowering core body temperature, and prompt application is lifesaving in managing exertional heat stroke. For in-match personal heat strain management (during changeovers and other brief breaks in play), the most practical and effective strategies include: using ice towels covering large skin surface areas, cold fluid ingestion (including slushies), shade or air-conditioned rest areas, and precooling with ice bath or cold showers before play begins or resumes. A 10-minute heat break has been shown to reduce core body temperature by ~0.25°C when athletes incorporate these strategies effectively.^[13]^ Intermittent or brief cooling is less effective than sustained cooling for at least 5–6 minutes. Safety is further enhanced by combining appropriate hydration with sodium replacement and structured cooling behaviours at changeovers and heat breaks.

**Brian Hainline:** Optimal cooling strategies integrate pre-match hydration and continuous thermal management during play. Players should begin competition in a euhydrated state and use each changeover for active cooling. Evidence-based methods include fluid intake at every changeover, use of shaded areas when possible, and application of cold or ice towels to the neck, head, and upper torso. If ice is unavailable, cold water poured over these regions remains effective. Regular towel-drying of sweat and clothing changes enhances evaporative efficiency. Collectively, these interventions reduce core temperature rise, sustain performance, and decrease risk of exertional heat illness.

**3. Rules and player groups:** **Should there be different heat policies for men, women, juniors, seniors, and wheelchair players? What differences would make sense?**

**Ollie Jay:** At the Australian Open, men and women in the main draw follow the same heat policy and environmental thresholds. Although women typically have a lower maximum cooling potential via evaporation, their metabolic heat production will also be lower.^[14]^ While minor differences exist in surface area-to-mass ratios between sexes, these are not significant enough to warrant separate risk thresholds. Recent evidence indicates that junior athletes aged 10 years and older show similar physiological responses to exercise in severe heat stress (up to 40°C) as adults, with comparable peak core temperatures and dehydration rates.^[15]^ However, to ensure duty of care, slightly lower environmental thresholds are advised for juniors reflecting their capacity to tolerate heat-related distress compared to professionals. For wheelchair players – particularly those with spinal cord injuries, whose sweating ability is diminished depending on injury severity – it is recommended that play be modified at even cooler and drier conditions.^[16]^

**Jelmer Alsma:** Different groups of players face different risks of heat stress, reflecting variations in thermoregulation and physiological reserves. Juniors have an immature sweating response, less effective heat dissipation, and a lower heat storage capacity. Seniors are more vulnerable due to reduced cardiovascular reserve and an altered muscle-to-fat ratio, which lowers body water and increases dehydration risk. Wheelchair players face specific challenges, such as impaired convective cooling and, in the case of spinal cord injury, reduced sweating below the lesion level. Although there are gender-related differences in thermoregulation, these do not result in substantial performance differences and are explained mainly by body composition, training status and acclimatisation.

Therefore, it is reasonable to implement differentiated heat policies for different groups, mitigating group-specific risks. These policies could include shorter formats, earlier suspension of play, longer breaks, and prioritised access to cooling methods and shaded rest areas. Overall, policies should prioritise safety for all players.

**Hein Daanen**: Paraplegic participants’ ability to thermoregulate is reduced, so it may be wise to take this into account when setting heat limits. Seniors have a reduced capacity to vasodilate and evaporate sweat, suggesting that lower thresholds may be appropriate. I am not sure about males/females and about healthy young children. A good literature review on this topic could help determine whether additional adjustments are warranted.

**Kathleen Stroia:** Heat policies should consider physiological and situational differences across groups. For example, ITF rules apply lower WBGT thresholds for wheelchair tennis (≥28°C modification, ≥30°C suspension) due to impaired thermoregulation and limited evaporation. Juniors require practical education and structured, appropriate scheduling with sufficient time between multiple same-day matches, as their physiology and risk are similar to adults, but their self-management is generally worse and repeated bouts are generally more demanding. Seniors may need earlier and more cautious interventions due to reduced cardiovascular and thermoregulatory efficiency. Men and women respond similarly physiologically, so differentiation is more about match format: women’s best-of-three matches typically allow breaks after set 2, men’s best-of-five after sets 3 or 4, but recommendations suggest aligning breaks more evenly. Close monitoring and safety should supersede tradition in all cases, with thresholds adjusted to group-specific risks and conditions.

**Brian Hainline:** Yes. Heat policies should be differentiated according to physiological and thermoregulatory capacity. Wheelchair athletes are particularly vulnerable due to potential impairments in sweating and autonomic regulation, necessitating longer recovery intervals and access to cooling facilities. Juniors and seniors exhibit less efficient thermoregulation and lower sweat rates, necessitating extended rest and hydration periods after two sets. Adult male and female players can adhere to comparable thresholds, with mandated breaks after two sets (or three in Grand Slams). Adapting policies by age, sex, and functional capacity reflects current scientific consensus on individualised risk and safety thresholds in hot environments.

**4. Preparation and acclimatisation:** **How can elite players realistically acclimatise to heat, given their travel schedules, and is this something tournaments should help support?**

**Jelmer Alsma:** It is often difficult for tennis players to fully acclimatise due to frequent travel, which can involve rapid climate changes. Full acclimatisation usually takes 7–14 days. During this process, players develop a faster and more effective sweat response, improved blood flow to the skin, normalisation of resting heart rate, a reduced core and skin temperature during exercise and, ultimately, better performance. Re-acclimatisation occurs more quickly after exposure, often within two to four days. Adaptations from heat carry over to cooler climates, but not vice versa. In practice, players can use heat acclimation protocols before tournaments or maintain ‘heat maintenance’ sessions between events. If direct acclimatisation is not possible, adaptation can be facilitated through post-exercise heat exposure (such as using a sauna or hot bath), passive heat exposure or training in extra clothing. Tournament organisers could facilitate this process by providing facilities for controlled heat exposure.

**Hein Daanen:** Players experience considerably reduced heat strain when acclimatised to hot conditions. Ideally, players should be aware that several days of training in the heat lead to better dry and wet (evaporative) heat loss. Also, the training schedule should be designed to minimise the decay of heat acclimation through regular exposure to hot conditions. Additionally, checking the specific gravity of the urine prior to matches or intense training and increasing fluid intake when dehydration is detected may help reduce the risk of kidney damage.

**Todd Ellenbecker:** Optimal acclimatisation is particularly challenging in professional tennis, given worldwide travel commitments and back-to-back tournament play. Research has consistently shown that optimally preparing and acclimating to an environment can take extended periods of time, which professional tennis players do not have. In contrast, extended acclimatisation periods are more feasible for junior players and lower-level competitors, who can compete in a series of tournaments within regions sharing similar temperature, altitude, humidity, and air quality. This consistency reduces the need for repeated adaptation. Although achieving full acclimatisation may not be possible, professional players—working closely with their physiotherapists, strength and conditioning staff and physiologists—should aim to arrive at tournament locations as early as practical to allow partial adaptation to local conditions. Furthermore, competing in multiple events under similar environmental conditions can enhance acclimatisation and support improved early performance.

**Kathleen Stroia:** Heat acclimatisation is one of the strongest protective factors, but elite players face ongoing challenges due to frequent global travel. Full acclimatisation (for those entirely unacclimatised to the heat) generally requires up to 10–14 days of progressive exercise-heat exposure, which is rarely feasible. Practical approaches include shorter acclimation periods (4–7 days), alternate heat exposure (indoor heat chamber, sauna, overdressing in training), and precooling strategies. Tournaments can support players by ensuring on- and off-court heat mitigation and readily accessible cooling facilities, such as on-court ice towels, shade, fans, off-court shaded or air-conditioned warm-up and recovery spaces, cool pools, and appropriate sports drinks and water. Coordinating tours and tournaments to enable adaptation and implementing heat rules, such as scheduling matches outside peak heat times, could lower the risk of exertional heat illness.

**Brian Hainline:** Elite tennis athletes face travel schedules that often undermine physiological recovery and heat adaptation. Ideally, acclimatisation requires 7–10 days of progressive exposure to the target environment, allowing cardiovascular, thermoregulatory, and perceptual adjustments. Tournament organisers and governing bodies share responsibility for facilitating acclimatisation through scheduling flexibility, early access to venues, and coordinated player education. Structural changes within the professional calendar are needed to align competition logistics with evidence-based recovery and acclimatisation protocols, thereby enhancing both performance and player safety.

**5. Medical treatment & prevention:** **How should exertional heat-related illnesses in tennis (cramps, heat exhaustion, heat stroke) be managed, and is hydration getting too much or too little emphasis in prevention?**

**Jelmer Alsma:** Prevention is key to managing heat-related illness. Although hydration is important, exertional heat stroke can also occur in well-hydrated athletes, and overhydration poses a risk for hyponatraemia. The most effective hydration strategy is personalised fluid and solute replacement based on individual sweat rate and sweat sodium concentration rather than generic drinking schedules. Other key preventive measures include heat acclimatisation and cooling strategies. Despite these measures, medical problems can still occur. Heat cramps can be managed through stretching, hydration and replacing electrolytes. Heat exhaustion requires rapid cooling, hydration and rest in a shaded and cool environment. Exertional heat stroke is a medical emergency with a core temperature > 40°C together with a lowered or altered mental status. Treatment aims to reduce core temperature to 39°C within 30 minutes through immediate, aggressive cooling — ideally whole-body cooling, or rotating ice towels if immersion is unavailable—before transport to hospital for further evaluation.

**Hein Daanen:** There is consensus on how to treat heat stroke: aggressive cooling, ideally in a cold-water bath. However, treating heat cramps or heat exhaustion is less clear in my view. Hydration status may be checked before games and training to avoid problems during matches.

**Todd Ellenbecker:** Player education is critically important from a prevention standpoint for these heat illnesses and should begin with both the medical team and the coaching staff. Players must understand the fundamental principles of hydration, including the importance of replacing BOTH fluid and electrolytes during practice and matches, ideally using consistent strategies that the body tolerates well. For players with a history of heat illness or reduced tolerance to challenging environmental conditions, testing hydration status and sweat concentrations may allow for more bespoke hydration planning. Such testing can be provided by federations and supported at tournaments by medical staff and nutritional consultants. Data collected over several years at numerous ATP tournaments consistently indicate that many players begin practice sessions already mildly or moderately dehydrated, often failing to adequately replace fluids lost during the previous day’s match or practice session. Therefore, combining objective monitoring with targeted educational initiatives is critical to preventing heat illness and maintaining player safety and performance.

**Kathleen Stroia:** Management should follow a “cool first, transport second” principle for suspected exertional heat stroke, with immediate cold-water immersion until core temperature < 39°C. Rectal thermometry is the gold standard for diagnosis and monitoring. Exertional muscle cramping (generally beginning with twitches and often wandering/spreading bilaterally) should be treated with stretching and individualised sodium/hydration strategies. Whereas appropriate and sufficient hydration is essential to performance and heat safety, exertional heat illness is multifactorial, primarily prompted by high-intensity, long-duration, and repeated exercise in challenging environmental conditions, with one’s risk also influenced by fatigue, acclimatisation status, health status (illness), and behavioural choices. Prevention involves personalised fluid and sodium management, proactive education, efficient preparation strategies, and pre-arranged tournament-level measures (heat breaks, shaded recovery, signage, staff training). Effective prevention balances hydration with cooling and acclimatisation, rather than focusing on fluid intake alone.

**Brian Hainline:** Hydration is essential but insufficient alone in preventing heat-related illness. Muscle cramps often reflect neuromuscular fatigue or electrolyte imbalance rather than dehydration per se. Heat exhaustion requires immediate medical evaluation, and continuation of play is generally contraindicated. Exertional heat stroke constitutes a medical emergency; evidence demonstrates 100% survival with rapid recognition and immediate whole-body cold-water immersion within 30 minutes (“cool first, transport second”). Tournaments must rehearse emergency protocols and ensure ice baths are available whenever ambient conditions pose risk. Prevention should emphasise comprehensive strategies: heat monitoring, workload modification, acclimatisation, and prompt medical readiness.

## Conclusion

Managing heat stress in tennis—no longer a side issue but a core safety concern—requires an integrated approach that aligns scientific evidence with on-court practice across all levels of play. This panel reaffirmed the continued value of WBGT as a practical decision-support tool for assessing heat stress, while also highlighting increasing momentum toward tennis-specific heat assessment models that more accurately capture the physiological and environmental demands of match and tournament play. Across player groups and competition settings, several consistent priorities emerged: evidence-based cooling strategies, appropriate differentiation of heat policies for vulnerable populations, proactive heat acclimatisation within the constraints of professional travel schedules, and robust medical preparedness, supported by appropriately trained staff, for the prevention and management of exertional heat illness.

Sustained progress will depend on strengthened collaboration and appropriate resources among researchers, clinicians, governing bodies, and tournament organisers to promote consistent policy development, equitable access to cooling and medical resources, and ongoing evaluation of emerging technologies and practices. By harmonising research, education, and operational decision-making, the sport is well positioned to advance a timely and more coherent global framework for heat management—one that safeguards player health while maintaining fairness and performance in an era of increasing environmental heat stress.

## References

[1] International Tennis Federation (2025). Men’s and Women’s Regulations 2025.

[2] International Tennis Federation (2025). ITF World Tennis Tour Juniors Regulations 2025.

[3] International Tennis Federation (2025). ITF World Tennis Masters Tour Regulations 2025.

[4] WTA Tour Inc (2025). 2025 WTA Official Rulebook.

[5] International Tennis Federation (2025). ITF Wheelchair Tennis Competition Regulations 2025.

[6] Chwasta M (2025). The Australian Open revamped its heat policy seven years ago: How does it work?.

[7] All England Lawn Tennis Club (2025). Heat rule: information about the heat rule used at the Championships.

[8] Chowdhury T (2025). What will the weather be like at the 2025 US Open?.

[9] Jay O, Vanos J, Gagnon D, Tartarini F To save lives in heatwaves, focus on how human bodies work. Nature 2025;.

[10] Lynch GP, Périard JD, Pluim BM (2018). Optimal cooling strategies for players in Australian Tennis Open conditions. J Sci Med Sport.

[11] Schranner D, Scherer L, Lynch GP (2017). In-play cooling interventions for simulated match-play tennis in hot/humid conditions. Med Sci Sports Exerc.

[12] Jay O, Morris NB (2018). Does cold water or ice slurry ingestion during exercise elicit a net body cooling effect in the heat?. Sports Med.

[13] Tippet ML, Stofan JR, Lacambra M, Horswill CA (2011). Core temperature and sweat responses in professional women’s tennis players during tournament play in the heat. J Athl Train.

[14] Gagnon D, Kenny GP (2012). Sex differences in thermoeffector responses during exercise at fixed requirements for heat loss. J Appl Physiol.

[15] Smallcombe JW, Topham TH, Brown HA (2025). Thermoregulation and dehydration in children and youth exercising in extreme heat compared with adults. Br J Sports Med.

[16] Forsyth P, Miller J, Pumpa K, Thompson KG, Jay O (2019). Independent influence of spinal cord injury level on thermoregulation during exercise. Med Sci Sports Exerc.

